# Responsibility for Dirty Milk

**Published:** 1923-10

**Authors:** 


### RESPONSIBILITY FOR DIRTY MILK.

A CORRESPONDENT in our September issue refers to the "unspeakably filthy conditions" under which he has seen cows being milked, and asks one or two plain questions to which plain answers can readily be given. "Who are the authorities responsible for seeing that milk is only taken from clean cows, milked with clean hands in clean cowsheds? And what are the precise powers of such authorities?" He also inquires why these authorities do not use their powers. Our correspondent is not alone in his impatience about pasteurization, grade A and the rest. At a recent conference the Medical Officer for the City of London (Dr. Howarth) referred to the seven different classes of milk that appeared to be recognised, and suggested that the last class should be "A.O.D.S." or "Any Other Dirty Stuff."

WHERE AUTHORITY RESTS.

We will deal with our correspondent's questions. The responsible authority in the country districts which he particularly has in mind is the Rural District Council. If the Council will not exercise their powers satisfactorily, those powers may, on complaint by the County Council, be transferred to the County authority. The powers that the Rural District Council may exercise include the making of regulations on these very essential matters of cleanliness. It is sufficient to quote two clauses of the Model Regulations issued by the Local Government Board (now the Ministry of Health):—

5. (1) Every cowkeeper shall cause every part of the interior of every cowshed in his occupation to be thoroughly cleansed from time to time as often as may be necessary to secure that such cowshed shall be at all times reasonably clean and sweet. 17. (5) He shall not cause or suffer any cow belonging to him or under his care or control to be milked for the purpose of obtaining milk for sale unless, at the time of milking, the udder and teats of such cow are thoroughly clean and unless the hands of the person milking such also are thoroughly clean and free from all infection and contamination.

"CONSERVATIVE AND OLD-FASHIONED METHODS."

In that mine of useful information, Robertson and Porter's "Sanitary Law and Practice," we read that "it is only within recent years that cowsheds have been given the serious consideration that their unwholesomeness demands. The majority of the cow-feeders of the British Isles are extremely conservative and greatly attached to old-fashioned methods. It is only the few who are willing to carry out improvements, to erect sheds of good design and construction and exercise care in handling the milk." Let us quote further from this book to see what is included in the euphemism about "conservative . . . old-fashioned methods." The processes of milking are dealt with in the following paragraph:—

Milkers are usually classified as dry and wet. The dry milker uses the hands in the ordinary condition; the wet milker, before attacking the teats, moistens the hands with water or milk or by spitting on them. Some persons use a lubricant such as vaseline. Wet milking is most objectionable, since there is always a risk of some of the liquid, mixed with the dirt from the hands and teats, dropping into the milk-pail and adding to the unnecessary contamination of its contents. Moreover, if the milker wishes further to moisten his hands in the course of the milking, he not infrequently dips them in the milk in the pail.

COUNTY COUNCIL INTERVENTION.

It is an unpleasing picture, that of the milker spitting on his doubtless dirty hands before he begins operations and moistening them at subsequent stages in the milk itself. Farmers, the older men particularly, are doubtless old-fashioned and conservative. But it will not do. We can probably find an answer to the inquiry of our correspondent as to the inaction of the authorities, merely by stating the fact that the farmers themselves are found sitting on the Rural District Councils. It is time that County Councils took a hand in the matter and that in every district there should be an enforcement of those elementary precautions which are observed where milch cows are properly kept by persons with adequate ideas of decency and of their responsibility to the community—clean hands, clean overalls, clean cows, clean utensils and clean cowsheds. Scientific inquiry and research in regard to milk are all to the good; but we can progress quite a long way within what has been called the Chadwick gospel of soap and water.

